# Hypoxic in vitro culture reduces histone lactylation and impairs pre-implantation embryonic development in mice

**DOI:** 10.1186/s13072-021-00431-6

**Published:** 2021-12-21

**Authors:** Wanting Yang, Peijun Wang, Pengbo Cao, Shuang Wang, Yuxiao Yang, Huimin Su, Buhe Nashun

**Affiliations:** State Key Laboratory of Reproductive Regulation and Breeding of Grassland Livestock, School of Life Sciences, Inner Mongolia University, Hohhot, 010070 China

**Keywords:** Histone lactylation, H3K23la, H3K18la, Oxygen, Hypoxia, Pre-implantation embryo, Embryonic development

## Abstract

**Background:**

Dynamic changes of histone posttranslational modifications are important contexts of epigenetic reprograming after fertilization in pre-implantation embryos. Recently, lactylation has been reported as a novel epigenetic modification that regulates various cellular processes, but its role during early embryogenesis has not been elucidated.

**Results:**

We examined nuclear accumulation of H3K23la, H3K18la and pan histone lactylation in mouse oocytes and pre-implantation embryos by immunofluorescence with specific antibodies. All of the three modifications were abundant in GV stage oocytes, and both H3K23la and pan histone lactylation could be detected on the condensed chromosomes of the MII oocytes, while H3K18la were not detected. After fertilization, the nuclear staining of H3K23la, H3K18la and pan histone lactylation was faint in zygotes but homogeneously stained both of the parental pronuclei. The signal remained weak in the early cleavage stage embryos and increased remarkably in the blastocyst stage embryos. Comparison of the embryos cultured in four different conditions with varying concentrations of oxygen found that H3K23la, H3K18la and pan histone lactylation showed similar and comparable staining pattern in embryos cultured in atmospheric oxygen concentration (20% O_2_), gradient oxygen concentration (5% O_2_ to 2% O_2_) and embryos obtained from in vivo, but the modifications were greatly reduced in embryos cultured in hypoxic condition (2% O_2_). In contrast, nuclear accumulation of H3K18ac or H3K23ac was not significantly affected under hypoxic condition. Moreover, the developmental rate of in vitro cultured embryo was significantly reduced by low oxygen concentration and small molecule inhibition of LDHA activity led to decreased lactate production, as well as reduced histone lactylation and compromised developmental rate.

**Conclusions:**

We provided for the first time the dynamic landscape of H3K23la, H3K18la and pan histone lactylation in oocytes and pre-implantation embryos in mice. Our data suggested that histone lactylation is subjected to oxygen concentration in the culture environment and hypoxic in vitro culture reduces histone lactylation, which in turn compromises developmental potential of pre-implantation embryos in mice.

**Supplementary Information:**

The online version contains supplementary material available at 10.1186/s13072-021-00431-6.

## Introduction

Gamete genomes undergo widespread epigenetic reprogramming to restore totipotency in zygotes [1, 2]. Covalent posttranslational modifications (PTMs) of histones, including methylation, acetylation, phosphorylation, ubiquitylation and sumoylation are essential content of epigenetic regulation [3–9], and play pivotal roles in zygotic genome activation and cell lineage specification during embryogenesis [10]. Recently, a lactate-derived lysine lactylation (Kla) has been proposed as a novel epigenetic modification, which is catalyzed by P300 and directly stimulates gene activation [11]. During the late phase of M1 macrophage polarization, histone lactylation regulates expression of homeostatic genes and promotes transformation of M2-like repair phenotype [11, 12]. Histone lactylation also promotes expression of macrophage pro-fibrotic genes in lung myofibroblast, and regulates key genes related to cell metabolism in non-small cell lung cancer cells [13, 14]. During somatic cell reprogramming, Gli-like transcription factor 1 (Glis1) activates glycolysis and elevates both histone acetylation and lactylation levels, which coordinately up-regulates expression of pluripotency genes to promote cellular reprogramming [15]. Apart from histone lactylation, global lysine lactylome analysis showed that lactylation in non-histone proteins regulates protein interactions in *Botrytis cinerea*, and is involved in central carbon metabolism and protein biosynthesis in *Oryza sativa* [16, 17]. Furthermore, lactylation has been shown to be involved in infection, cancer, differentiation and biosynthesis, but its role during fertilization and early embryogenesis has not been elucidated [18].

The development of pre-implantation embryos depends on appropriate reproductive tract environment [19], of which oxygen is an essential component. Oxygen plays important roles in embryonic development by regulating gene expression and metabolism [20]. The oxygen concentration is 5–8% in the fallopian tube, and 1.5–3.5% in the uterus [21]. However, in vitro culture of embryo is generally conducted in the atmospheric oxygen concentration (~ 20%), which differs from the oxygen concentration in the reproductive tract in vivo [22, 23]. At early cleavage stages, embryos mainly rely on oxidation of pyruvate for energy supply [19, 24], while in post-compaction embryos, glucose metabolism increases significantly and becomes the predominant energy source in the blastocyst stage embryos [25]. In mouse embryo, blastocyst stage embryos exhibit high glucose uptake and high oxygen consumption [19]. Under hypoxic oxygen concentration, the oxidation rate of glucose increases in blastocysts, while the glycolysis rate decreases in contrast, which results in reduced production of lactate [26].

Oxygen concentration regulates early embryonic development through fine-tuning expression of important transcription factors and genes related to glycolysis pathway [27]. Cellular response to low oxygen condition is mainly mediated by the activation of the oxygen sensitive transcription factors, Hypoxia Inducible Factors (HIFs) [28]. HIFs modulate expression of glucose transporters and glycolytic enzymes [29]. Glucose transporters such as GLUT1, GLUT2, and GLUT3 are critical for early embryonic development. GLUT-3 mediates uptake of glucose into the blastocyst from the external environment, while GLUT-1 is responsible for glucose efflux into the blastocoel cavity [30]. It has been reported in bovine embryo that oxygen regulates expression pattern of the GLUT1 through HIF2α [31], and 2% oxygen concentration alters the expression of GLUT-1, GLUT-3 and VEGF in the post-compaction mouse embryos [32].

In this study, we characterized the nuclear accumulation of H3K18la, H3K23la and pan histone lactylation in oocytes, zygotes and pre-implantation embryos in mice for the first time, and further explored the relationship between oxygen concentration and the lactylation modifications in pre-implantation embryos. Inhibition of the Lactate dehydrogenase A (LDHA) activity reduced histone lactylation level and impaired developmental rate of the pre-implantation embryos. Our findings suggested that hypoxic in vitro culture reduces histone lactylation, which in turn impairs pre-implantation development in mice.

## Result

### Nuclear accumulation of the histone lactylation in pre-implantation embryos

**Fig. 1:**
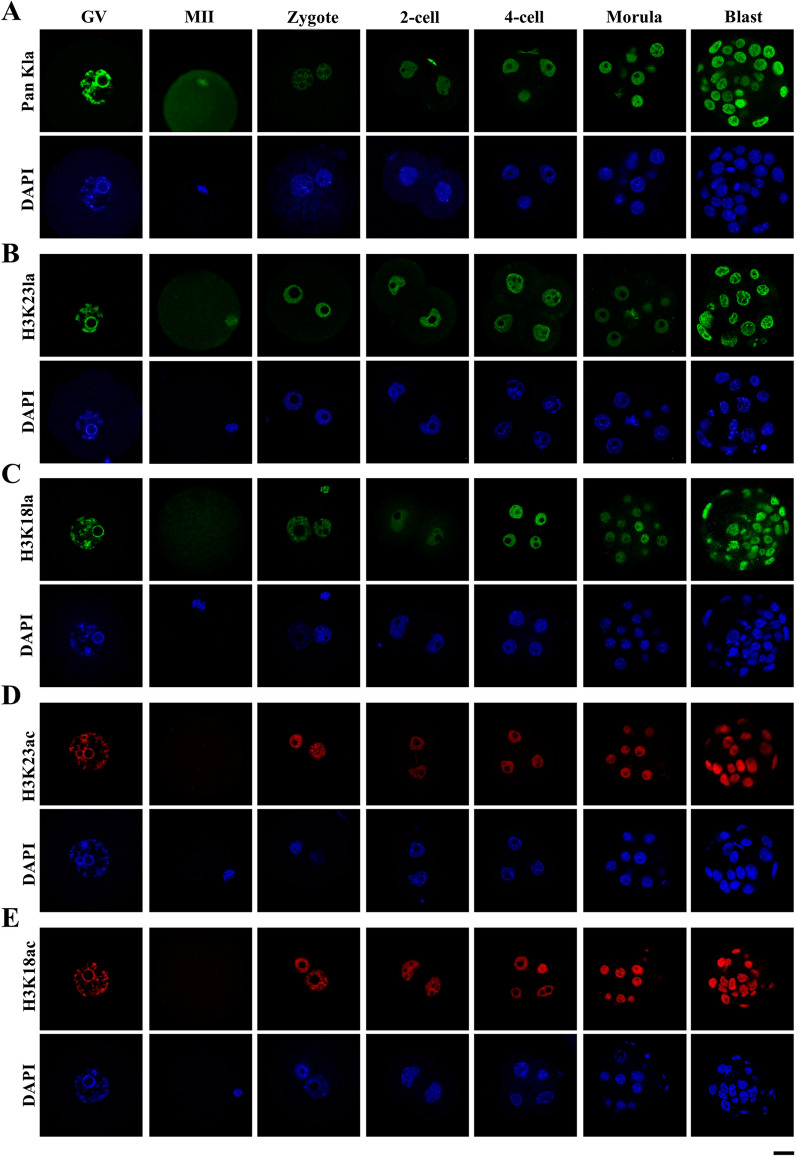
Nuclear accumulation of histone lactylation and histone acetylation in mouse oocytes and pre-implantation embryos. **a**–**e** Histone lactylation and histone acetylation were detected in mouse oocytes and pre-implantation embryos. Specific antibodies were used to detect the pan histone lactylation (**a**), H3K23la (**b**), H3K23ac (**c**), H3K18la (**d**), H3K18ac (**e**) in mouse oocytes at the germinal vesicle (GV) and metaphase II (MII) stages, as well as pre-implantation embryos at the zygote (zygote), 2-cell (2-cell), 4-cell (4-cell), morula (Morula) and blastocyst (Blast) stages. Pan histone lactylation, H3K23la and H3K23ac were shown in green. H3K18la and H3K18ac were shown in red. The DNA was stained with DAPI (Blue). More than 9 embryos were examined in each stage of each condition. Scale bars: 20 μm

Histone lysine lactylation (Kla) as a novel epigenetic modification has been recently reported to play important roles in different cellular processes [11], and we set out to examine the dynamic distribution of histone lactylation in oocytes and early embryos in mouse. First, we examined distribution of pan histone lysine lactylation, histone H3 lysine23 lactylation (H3K23la) and histone H3 lysine 18 lactylation (H3K18la) using specific antibodies in oocytes and in vitro fertilized and cultured embryos, respectively. In the germinal vesicle (GV) stage of fully grown oocytes, strong immunofluorescence signals of pan histone lactylation, H3K23la and H3K18la were observed (Fig. 1A–C). When the oocytes develop to mature metaphase II (MII) stage, pan histone lactylation and H3K23la were detected from the condensed chromosomes (Fig. 1A, B), but not the H3K18la (Fig. 1C). After fertilization, weak but distinct pan histone lactylation signal was detected from the paternal and maternal pronuclei in zygotes. The signal intensity gradually increased with embryonic development and reached the highest level in the blastocyst stage embryos (Fig. 1A). In contrast, H3K23la and H3K18la were constantly stained in the early cleavage stage embryos and the fluorescence intensity increased to the maximum in the blastocysts (Fig. 1B, C). In comparison, we also examined the nuclear accumulation of H3K23ac and H3K18ac during pre-implantation development, which showed relatively constant staining (Fig. 1D, E). In the GV stage, the oocyte nucleus showed strong H3K23ac and H3K18ac immunofluorescence signals, which was disappeared in the MII stage, and re-appeared after fertilization (Fig. 1D, E).

It has been well documented in mammalian zygotes that the paternal and maternal genomes remain physically separated after fertilization [33] and epigenetic asymmetry for DNA methylation, H3K9me2, H3K27me3, and H3K9ac exists between the male and female pronuclei [34, 35]. To test if it is also the case for histone lactylations, we examined the zygotes at 2, 4, 6, 8 and 10 hpi (hour post-insemination). Paternal and maternal pronuclei were all homogeneously stained with pan histone lactylation, H3K23la and H3K18la at different pronuclear stages of zygotes, except from the absence of H3K18la from the parental genome just after fertilization around 2 hpi (Additional file 1: Fig. S1A–C). These results demonstrated the nuclear presence of pan histone lactylation, H3K23la and H3K18la in oocytes and pre-implantation embryos and showed that there was no asymmetry for histone lactylation between male and female pronuclei in the zygotes. Similarly, we also examined H3K23ac and H3K18ac in the zygotes at 2, 4, 6, 8, and 10 hpi (Additional file 1: Fig. S1D, E). Interestingly, while H3K23ac stained both of the parental pronuclei, H3K18ac was only detected from the paternal genome in the zygotes around 2 hpi. From then on, both H3K23ac and H3K18ac were homogeneously distributed on parental pronuclei (Additional file 1: Fig. S1D, E). These data collectively showed that although the kinetics of initial establishment on parental genome is different, H3K23la, H3K23ac, H3K18la, H3K18ac and pan histone lactylation were present in the genome during the entire pre-implantation embryonic development.

### Histone lactylation in pre-implantation embryos was correlated with oxygen concentrations in the culture environment

**Fig. 2:**
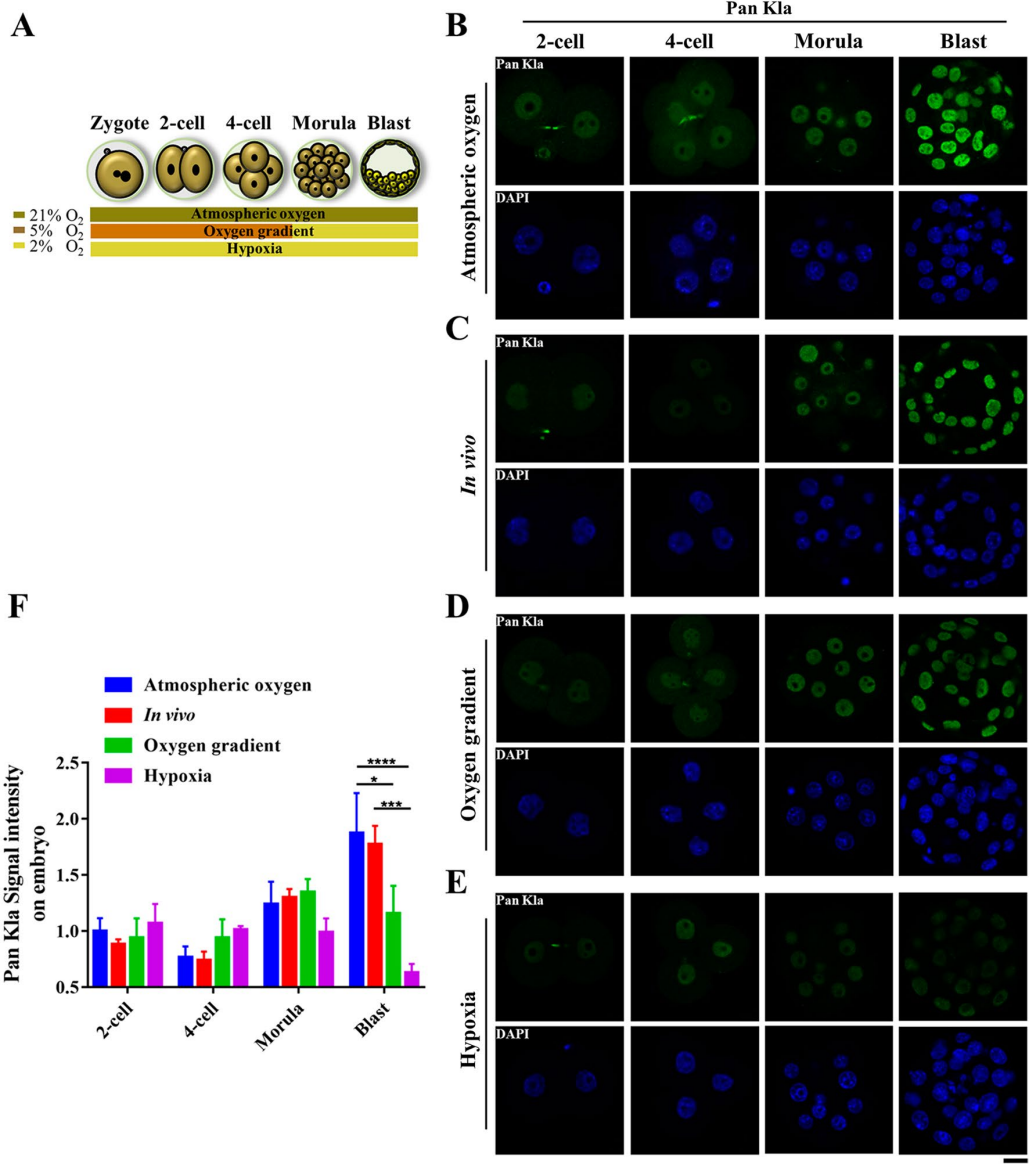
Dynamics of pan histone lactylation in pre-implantation embryo under different oxygen concentrations. **a** Schematic illustration of different oxygen conditions during in vitro embryonic culture. **b**–**e** Hypoxia reduced the pan histone lactylation in blastocysts. Pan histone lactylation (Green) in pre-implantation embryos at 2-cell (2-cell), 4-cell (4-cell), morula (Morula), and blastocyst (Blast) stages collected from the atmospheric oxygen group (**b**), in vivo group (**c**), oxygen gradient group (**d**) and hypoxia group (**e**) was examined. DNA was stained with DAPI (Blue). More than 10 embryos were examined in of each stage each condition. Scale bars: 20 μm. **f** Quantification of the fluorescence intensity for pan histone lactylation in embryonic blastomeres (*n* = 30 blastomeres per developmental stage in different oxygen conditions, 3 independent experiments). Error bars indicated SEM. Statistical analysis was carried out using two-way ANOVA followed by Tukey's multiple comparisons test. ⁎*p* < 0.05; ⁎⁎⁎*p* < 0.001; ⁎⁎⁎⁎*p* < 0.0001

**Fig. 3:**
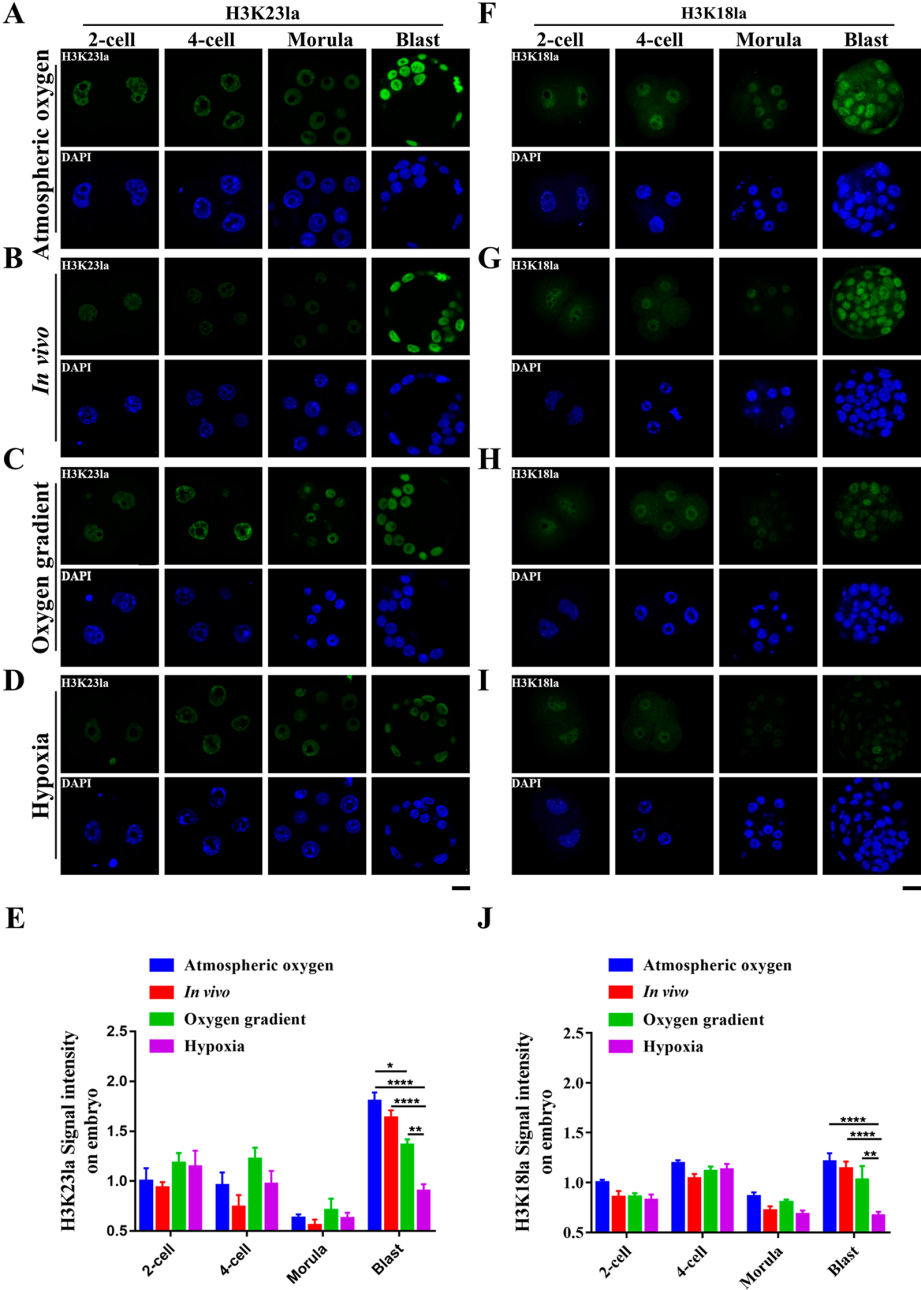
Nuclear accumulations of H3K23la and H3K18la in pre-implantation embryos under different oxygen conditions. **a**–**d** Long-term hypoxic in vitro culture significantly reduced H3K23la in blastocyst stage embryos. Immunofluorescence staining for H3K23la (Green) in mouse embryos at the 2-cell (2-cell), 4-cell (4-cell), morula (Morula) and blastocyst (Blast) stages embryos collected from the atmospheric oxygen (**a**), in vivo (**b**), oxygen gradient (**c**) and hypoxia (**d**) groups. DNA was stained with DAPI (Blue). **e** Quantification of H3K23la fluorescence intensity in embryonic blastomeres (*n* = 30 blastomeres per group, 3 independent experiments). **f**–**i** Level of nuclear H3K18la was significantly reduced in blastocyst stage embryos under long-term hypoxic in vitro culture conditions. Immunofluorescence staining for H3K18la (Green) in mouse embryos at the 2-cell (2-cell), 4-cell (4-cell), morula (Morula) and blastocyst (Blast) stages embryos collected from the atmospheric oxygen (**f**), in vivo (**g**), oxygen gradient (**h**), and hypoxia (**i**) groups. DNA was stained with DAPI (Blue). More than 12 embryos were examined in each stage of each condition. Scale bars: 20 μm. **j** Quantification of H3K18la fluorescence intensity in embryonic blastomeres (*n* = 30 blastomeres per group, 3 independent experiments). Error bars indicated SEM. Statistical analysis was carried out using two-way ANOVA followed by Tukey's multiple comparisons test. ⁎*p* < 0.05; ⁎⁎*p* < 0.01; ⁎⁎⁎*p* < 0.001; ⁎⁎⁎⁎*p* < 0.0001

Oxygen has been shown to regulate energy metabolism and gene expression during pre-implantation embryo development [31]. Lactate, as an important metabolite in early embryos, is regulated by oxygen, but modulates histone lactylation by itself [11, 26]. To explore the effects of different oxygen concentrations on histone lactylation in early pre-implantation embryos, we compared embryos cultured in or obtained from four different conditions: atmospheric oxygen group, in vivo group, oxygen gradient group and hypoxia group (Fig. 2A). Immunofluorescence staining with specific antibodies showed that there was no obvious difference in pan histone lactylation level in the early cleavage stage embryos in all groups (Fig. 2B–E). At blastocyst stage, the pan histone lactylation in the atmospheric oxygen group was similar to that in the in vivo group, but the pan histone lactylation in the oxygen gradient group and the hypoxia group was obviously lower, especially in the hypoxia group (Fig. 2B–E). Quantification of the fluorescence intensity showed that the pan histone lactylation levels in the oxygen gradient group and the hypoxia group were significantly lower than that of the atmospheric oxygen group or in vivo group only at blastocyst stage (Fig. 2F). Likewise, no significant change was observed for H3K23la and H3K18la until the blastocyst stage in all groups (Fig. 3). In the blastocysts, lower levels of H3K23la (Fig. 3A–D) or H3K18la (Fig. 3F–I) were observed in the oxygen gradient group and hypoxia group, with even marked reduction in the hypoxia group. Quantitation of the H3K23la and H3K18la immunofluorescence intensity showed that H3K23la and H3K18la were reduced only in blastocyst stage embryos in the oxygen gradient group and the hypoxia group compared to the atmospheric oxygen group or in vivo group (Fig. 3E, J). In contrast, nuclear accumulation of histone acetylation was not affect by different oxygen concentration. The staining intensities of H3K23ac (Additional file 2: Fig. S2A–D) and H3K18ac (Additional file 2: Fig. S2E–H) were almost constant throughout pre-implantation embryonic development and comparable between the same developmental stages of embryos collected from the four different conditions.

**Fig. 4:**
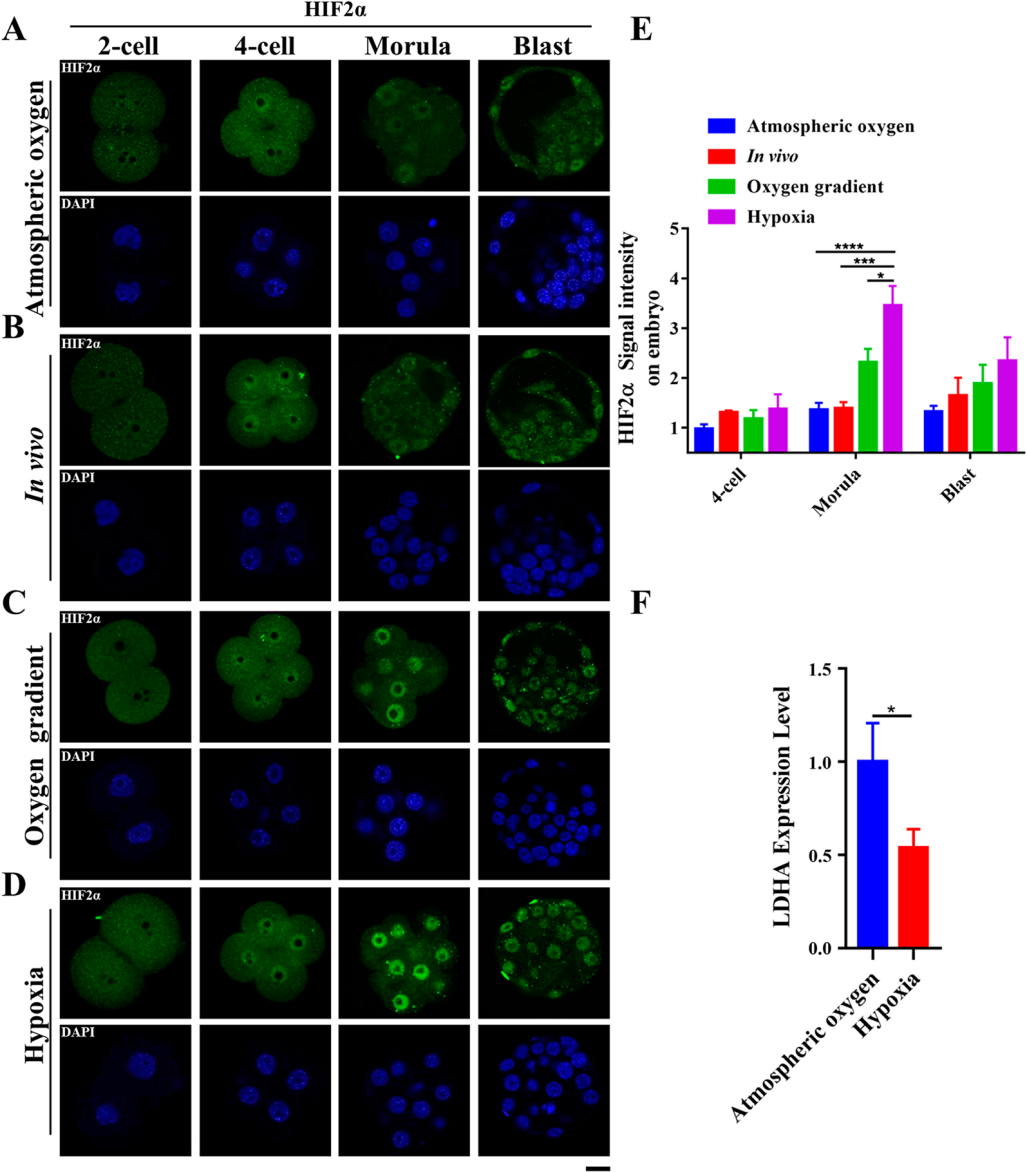
Nuclear accumulation of HIF2α in pre-implantation embryo under different oxygen concentrations. **a–d** Detection of HIF2α (Green) in pre-implantation embryos at 2-cell (2-cell), 4-cell (4-cell), morula (Morula), and blastocyst (Blast) stages collected from atmospheric oxygen (**a**), in vivo (**b**), oxygen gradient (**c**) and hypoxia (**d**) groups. DNA was stained with DAPI (Blue). More than 8 embryos were examined in each stage of each condition. Scale bars: 20 μm. **e** Quantification of fluorescence intensity for HIF2α in embryonic blastomeres (*n* = 30 blastomeres per developmental stage in different oxygen conditions, 3 independent experiments). Error bars indicates SEM. Statistical analysis was carried out using two-way ANOVA with Tukey's multiple comparisons test. **f** Hypoxic in vitro culture reduced expression of LDHA in blastocyst stage embryos. Relative expression level of LDHA was examined by quantitative real-time PCR in blastocyst stage embryos collected from atmospheric oxygen group and hypoxia group (*n* = 60 embryos in each group, 3 independent experiments). The mean value for blastocysts cultured in atmospheric oxygen group was set as 1, and relative values for hypoxia group samples were calculated accordingly. Error bars represented SEM. Statistical analysis was performed using two-tailed unpaired Student’s *t* test. ⁎*p* < 0.05; ⁎⁎*p* < 0.01; ⁎⁎⁎*p* < 0.001; ⁎⁎⁎⁎*p* < 0.0001

To confirm the hypoxic culture conditions, we used specific antibodies to examine the expression of the well characterized hypoxia inducible genes, HIF1α and HIF2α [62], in the pre-implantation embryos. Although readily detected in GV stage oocytes, HIF1α was not detected after fertilization in embryos neither in the atmospheric oxygen group nor in the hypoxia group (Additional file 3: Fig. S3A, B). In contrast, although totally absent from 2-cell embryonic nucleus in all groups, faint but distinct nuclear staining of HIF2α was first detected in the 4-cell stage embryos (Fig. 4A–D). While the staining intensity remained weak in the morula band blastocyst stage embryos in the atmospheric oxygen group and in vivo group (Fig. 4A, B), the nuclear staining signal of HIF2α was increased remarkably in the morula stage embryos both in the oxygen gradient group and the hypoxia group, and declined in the blastocyst stage embryos (Fig. 4C, D). Quantification of the fluorescence intensity showed that HIF2α was significantly increased in the morula stage embryos in the oxygen gradient group and the hypoxia group compared with the atmospheric oxygen group and the in vivo group (Fig. 4E), suggesting that the pre-implantation embryos responded to the low oxygen condition through activating hypoxia inducible gene HIF2α.

Glycolysis in the pre-implantation embryo is regulated by oxygen concentration [24] and LDHA catalyzes the conversion of pyruvate to lactate in glycolysis. Since lactate is an important regulator of histone lactylation [11, 39], we compared the expression of LDHA in blastocysts cultured under atmospheric oxygen or hypoxic conditions. RT-qPCR results showed that the expression of LDHA in the blastocysts cultured at hypoxic condition (2% O_2_) is significantly lower than that of atmospheric oxygen concentration (~ 20% O_2_) (Fig. 4F), indicating that LDHA expression was decreased under low oxygen concentration. Therefore, it is indicating a possibility that hypoxic culture leads to lower LDHA expression, which presumably contributes partially to the reduced lactate production and decreased histone lactylation.

### The effect of different oxygen concentrations on pre-implantation embryonic development

**Fig. 5:**
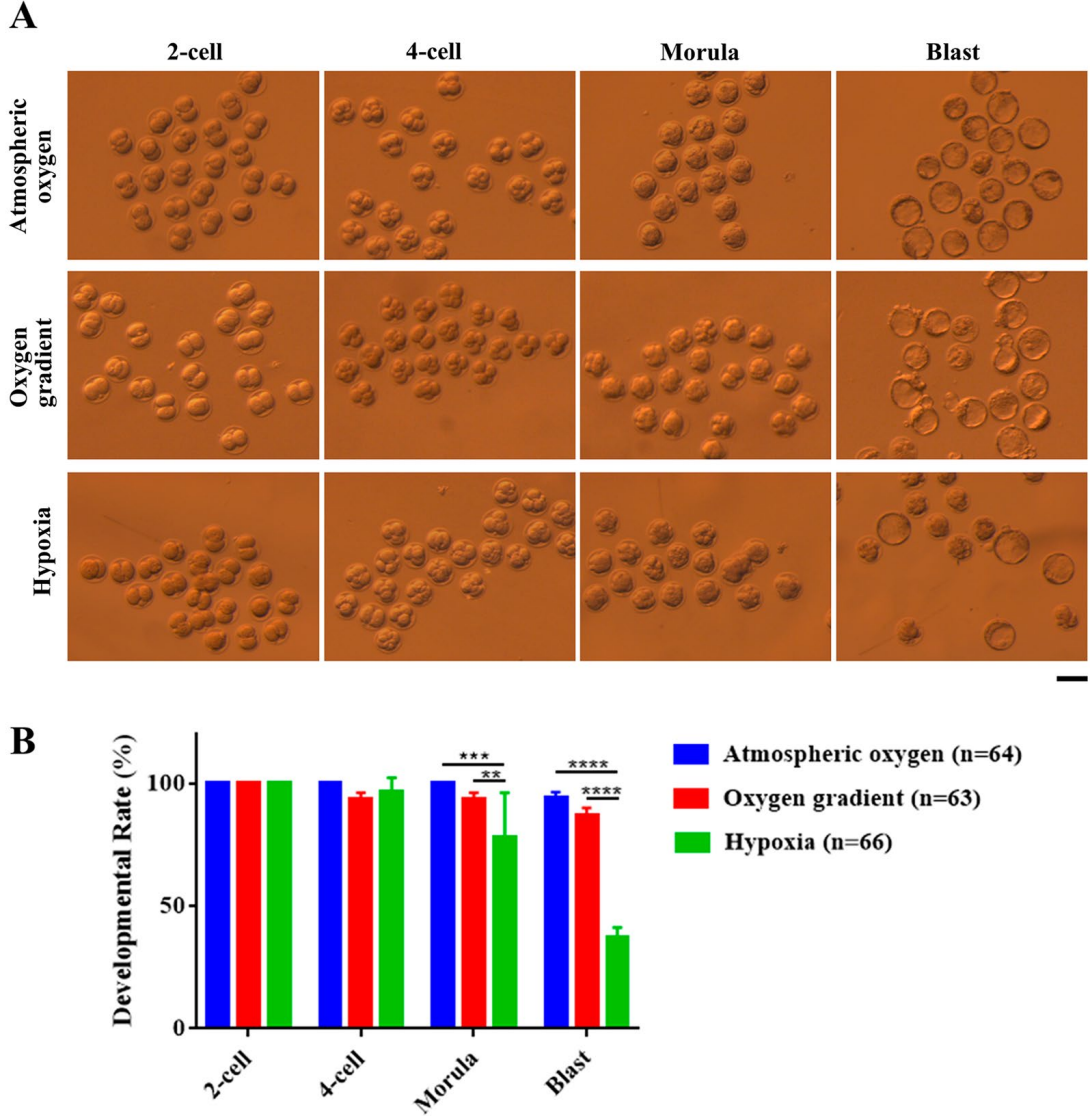
Effect of lower oxygen conditions on pre-implantation embryonic development cultured in vitro. **a** Representative images of pre-implantation embryos at 2-cell (2-cell), 4-cell (4-cell), morula (Morula), and blastocyst (Blast) stages in the atmospheric oxygen, oxygen gradient and hypoxia groups. Scale bars: 50 μm. **b** Quantification of developmental rate from 2-cell to blastocyst stage (n = 64 in atmospheric oxygen group; n = 63 in oxygen gradient group; n = 66 in hypoxia group, 3 independent experiments). The mean value for 2-cell was set as 1 and relative values for all other samples were calculated accordingly. Data are presented as mean ± SEM. Statistical analysis was carried out using two-way ANOVA followed by Tukey's multiple comparisons test. ⁎⁎*p* < 0.01; ⁎⁎⁎*p* < 0.001; ⁎⁎⁎⁎*p* < 0.0001

To investigate the effect of oxygen concentrations on embryonic development, in vitro fertilized oocytes were cultured under different oxygen concentrations as shown in the Fig. 2A. To assess the developmental capacity, 4-cell, morula and blastocyst stage embryos were examined morphologically at 48 hpi, 72 hpi and 96 hpi, respectively, and the developmental rates were calculated relative to the number of 2-cell stage embryos. Embryonic development was not affected by the different oxygen concentrations until the morula stage. All or majority of the embryos developed to the morula stage in the atmospheric oxygen group (100%) and the oxygen gradient (~ 94%) group, but the developmental rate decreased significantly in the hypoxia group (~ 78.0%) at morula stage (Fig. 5A, B), which was even pronounced at the blastocyst stage. While ~ 94% and ~ 87% of embryos developed to the blastocyst in the atmospheric oxygen group and in the oxygen gradient group, respectively, only ~ 37% of the embryos in the hypoxia group developed to the blastocyst (Fig. 5A, B). These results suggested that long-term hypoxia after fertilization had a detrimental effect on developmental potential of the pre-implantation embryonic development.

### Inhibition of LDHA reduced histone lactylation and developmental rate in early pre-implantation embryo

**Fig. 6:**
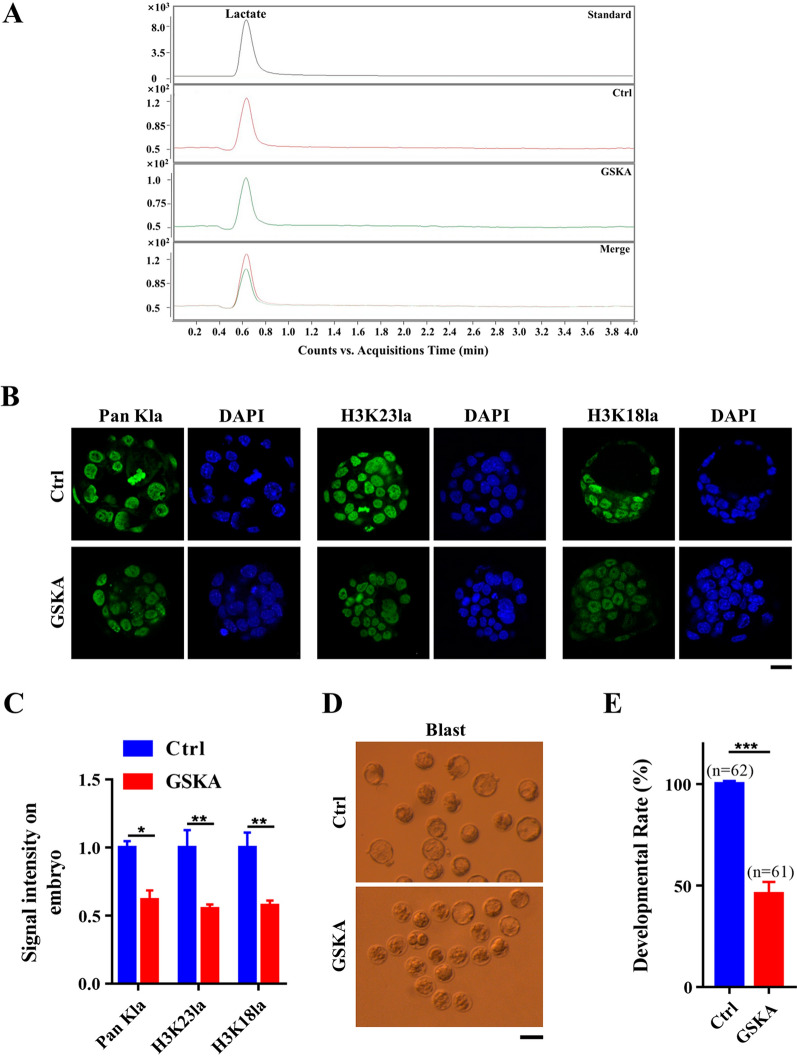
Inhibition of LDHA activity was detrimental for pre-implantation embryonic development. **a** UPLC–MS chromatogram of endogenous lactate extracted from NIH3T3 cells after 48 h treatment with DMSO or GSKA. **b** GSKA treatment led to reduced pan histone lactylation, H3K23la and H3K18la in the blastocysts. Morula stage embryos (72 hpi) were cultured with 100 pmol of GSKA (LDHA inhibitor) or the same concentration of DMSO (Ctrl) for 1 h, then transferred to fresh medium and cultured till blastocyst stage. Representative immunofluorescence staining of the pan histone lactylation (Green), H3K23la (Green) and H3K18la (Green) in blastocyst stage embryos were shown. DNA was stained with DAPI (Blue). Scale bars: 20 μm. **c** Quantification of fluorescence intensity for pan histone lactylation, H3K23la and H3K18la in embryonic blastomeres (*n* = 30 blastomeres per group, 3 independent experiments). **d** Representative images of blastocysts (Blast) treated with DMSO (Ctrl) or GSKA (GSKA). More than 10 embryos were examined in each stage of each condition. Scale bars: 50 μm. **e** Quantification of developmental rate of embryos treated with DMSO (Ctrl) or GSKA (GSKA) at the morula stage (*n* = 62 cells in ctrl group; *n* = 61 cells in GSKA group, 3 independent experiments). Error bars indicated SEM. Statistical analysis was performed using two-tailed unpaired Student’s t test. ⁎*p* < 0.05; ⁎⁎*p* < 0.01; ⁎⁎⁎ *p* < 0.001

To examine the relationship between lactate production and histone lactylation, we inhibited LDHA activity using GSK2837808A (GSKA) [36], a specific and effective small molecule inhibitor, which can reduce the production of lactate in glycolysis. First, we performed dose finding trials of GSKA in pre-implantation embryos. 2-cell (24 hpi), 4-cell (48 hpi) and morula (72 hpi) stage embryos were treated with 100 nmol, 10 nmol, 1 nmol, 100 pmol or 10 pmol of GSKA for 1 h, 2 h, 4 h, or 6 h, respectively, and continued to culture in drug-free medium. Control embryos were treated with the same concentration of DMSO. We found that 1 h treatment of morula stage embryos with 100 pmol of GSKA significantly inhibited the blastocyst formation rate (Additional file 4: Fig. S4A). Higher treatment concentration or longer treatment time almost completely inhibited embryonic development, while lower concentration did not have significant effect (Additional file 4: Fig. S4A). Therefore, 1 h incubation of morula stage embryos with 100 pmol of GSKA was determined to be the most suitable treatment in embryos. To further verify the inhibitory effect, NIH3T3 cells were incubated with 100 pmol of GSKA for 48 h and the content of intracellular lactate was examined by UPLC-ESI–MS. Average cellular lactate content was 269.0 ± 4.7 ng/mL (*n* = 3) in the GSKA treated group, which was reduced by 7.1% compared to the control group (289.5 ± 2.4 ng/mL, *n* = 3) (Fig. 6A), demonstrating the effective inhibitory effect of GSKA for LDHA activity. Therefore, morula stage embryos were treated with 100 pmol GSKA or the same concentration of DMSO for 1 h, and pan histone lactylation, H3K23la and H3K18la was examined in the blastocyst stage embryos (Fig. 6B). Immunofluorescence staining and quantification of fluorescence intensity showed that GSKA treatment reduced the pan histone lactylation, H3K23la and H3K18la by 38%, 51% and 43%, respectively (Fig. 6C). In addition, the developmental rate of blastocysts was reduced by 54% after GSKA treatment (Fig. 6D, E). These results suggested that 100 pmol of GSKA effectively inhibited LDHA activity and led to reduced production of lactate, which in turn led to lower histone lactylation and impaired embryonic developmental.

## Discussion

Epigenetic dynamics regulates gene expression in a spatial and temporal manner to safeguard developmental potential and cell lineage specification in the pre-implantation embryos [37, 38]. Lactylation is a recently discovered epigenetic modification which mainly happens on histone proteins [13, 39] and has been shown to play important roles in regulation of gene expression, somatic cell reprogramming and tumorigenesis [11, 15, 39]. Currently, 26 and 16 core histones lactylation sites have been identified in human MCF-7 cells and mouse bone-marrow-derived macrophage, respectively [11], among which H3K23la and H3K18la are common modifications both in mice and humans [11, 39, 40]. Here, we examined the dynamics of H3K23la, H3K18la and pan histone lactylation, in comparison with H3K23ac and H3K18ac, and explored their possible correlation with oxygen concentration in pre-implantation embryos in mouse. Though histone acetylation on H3K23 and H3K18 remained constant (Fig. 1D, E), all of three histone lactylation increased sharply in the blastocyst stage embryos (Fig. 1A–C). While pyruvate is the main energy supply in the early cleavage stage embryos, lactate produced during glycolytic metabolism serves as the main energy source at post-compaction stage [19, 41]. Since lactate level reaches the maximum in the blastocyst stage embryos [42] and histone lactylation is specifically modulated by lactate [11], the increased histone lactylation might partially due to the abundant lactate level in the blastocyst stage embryos (Fig. 1A–C). LDHA is the main enzyme for lactate production in glycolysis and when LDHA activity was inhibited by small molecule inhibitor GSKA, H3K23la, H3K18la and pan histone lactylation were all significantly reduced in blastocyst (Fig. 6B, C), supporting the hypothesis that histone lactylation is closely correlated with lactate level in mouse embryos.

Oxygen plays an important role in embryonic development by regulating epigenetic dynamics and energy metabolism [37, 43]. Hypoxic condition increases lactate production in somatic cells [44], however, that is not the case in embryos, especially in post-compaction stage [26]. Glucose is the primary nutrient of the blastocyst and is metabolized both oxidatively and through glycolysis [25]. Lactate is one of the end products of glycolysis, and a lower glycolysis rate results in decreased lactate production. Indeed, in post-compaction embryos, the glycolysis rate is reduced by 25% under hypoxic conditions and more glucose is oxidized, leading to reduced lactate production [25, 26, 45]. Therefore, the obviously reduced histone lactylation in blastocysts (Figs. 2, 3) might due to the reduced lactate level. In support of this view, LDHA expression was also reduced under low oxygen concentration compared with the atmospheric oxygen concentration in the blastocyst stage embryos (Fig. 4F), indicating that lactate production is impaired in hypoxic condition. However, in vitro fertilization and subsequent embryonic culture are generally carried out at atmospheric oxygen concentration (~ 20% O_2_) [46], and we did not observe obvious difference in histone lactylation between atmospheric oxygen group and in vivo group (Figs. 2B, C, 3A, B, F, G). The in vivo oxygen concentration is much lower than atmospheric oxygen concentration [47, 48]. However, the HIF2α that is often induced in response to low oxygen level is rarely expressed in embryos obtained from in vivo, just like the embryos cultured in vitro under atmospheric oxygen concentration (Fig. 4A, B). Intriguingly, the blastocysts obtained from in vivo and in vitro have very similar gene expression patterns [49], indicating that in addition to the oxygen, other variables in the culture environment might regulate gene expression and embryonic development [50, 51]. Indeed, lactate production in embryos is also regulated by some other factors, including glucose concentration and the composition of amino acids in the culture medium [52, 53]. Therefore, the comparable embryonic histone lactylation between the atmospheric oxygen group and the in vivo group may be the result of a combinatory regulation of multiple factors including oxygen. Notably, hypoxic condition did not significantly alter acetylation level of H3K23ac and H3K18ac (Fig. 3 and Additional file 2: Fig. S2), indicating the functional difference of histone acetylation and histone lactylation during pre-implantation embryonic development.

When transferred from 7 to 2% oxygen concentration at morula stage and cultured onward, the proportion of inner cell mass cells was increased in bovine blastocyst stage embryos [31]. Similarly, when human zygotes were cultured in 5% oxygen concentration until 4-cell stage and then changed to 2% oxygen concentration and cultured continuously, the yield and quality of blastocysts were improved significantly [54]. In contrast, when mouse embryos were first cultured under 7% oxygen concentration till morula stage and then at 2% oxygen concentration to the blastocyst stage, the developmental rate was not affected [55]. In line with these findings, when we changed the oxygen concentration from 5 to 2% at 4-cell stage embryo, the subsequent progression to blastocyst stage was not altered (Fig. 5A, B). However, the developmental potential was significantly impaired when the embryos were cultured entirely in 2% oxygen concentration from zygote to blastocyst (Fig. 5A, B). These findings collectively suggested that although short exposure to low oxygen concentration does not impair embryonic development, longer in vitro culture under 2% of oxygen is detrimental for the pre-implantation embryonic development in mice. It seems likely that the reduced developmental potential is, at least in part, due to the lower histone lactylation. Since histone lactylation is a crucial regulator of gene expression [11, 13–15], the reduced histone lactylation may interfere with normal embryonic genes expression and impairs developmental potential.

Accumulating evidences showed that crosstalk between metabolism and epigenetics plays important roles in gene expression, cell proliferation and cell differentiation [56], and has been attracting increasing interests in the context of development [57]. Recently, it was reported that high level of α-Ketoglutarate, an intermediary metabolite in the TCA cycle, regulates DNA methylation/demethylation dynamics through TET activity to promote pluripotency and improve embryonic development [58, 59]. Here, we propose that lactate, as an important cellular metabolite, not only provides energy for post-compaction embryonic development [25], but also regulates dynamics of histone lactylation [11]. Hypoxic in vitro culture leads to reduced lactate production and lower histone lactylation, which potentially disturb normal expression of genes [11, 15] and compromises developmental potential in pre-implantation embryos. Our findings provide a novel example of metabolic–epigenetic interplay in mouse pre-implantation embryos and are of great significance for improving in vitro embryonic culture. However, the underlying molecular mechanism how oxygen regulates lactate production and histone lactylation, as well as how histone lactylation fine-tunes gene expression during pre-implantation embryos remains to be elucidated.

## Conclusions

In this study, we provided for the first time the nuclear staining landscape of histone lactylation in oocytes and pre-implantation embryos in mice. Pan histone lactylation, H3K23la, and H3K18la were all abundant in oocytes and pre-implantation embryos, and reach the highest level in the blastocyst stage embryos, suggesting their potential involvement during pre-implantation development. Our data also suggested that histone lactylation is correlated with cellular lactate level in the embryos and subjected to variation of oxygen concentration in the culture environment. Moreover, hypoxic in vitro culture reduces histone lactylation and compromises developmental potential of pre-implantation embryos.

## Methods

### Mice

ICR mice used for oocytes/embryos collection were purchased from Vital River Laboratory (Beijing, China), and were housed and bred in a specific pathogen-free facility in the Inner Mongolia University. The mice were kept at a constant temperature of 22 ± 2 °C on a 12 h light/dark cycle and had unrestricted access to chow and water.

### Oocytes/embryos collection, in vitro fertilization (IVF) and assessment of early embryonic development

Female ICR mice, ~ 8 weeks, were super-ovulated by intraperitoneal (i.p.) injection of 10 IU pregnant mare serum gonadotropin (PMSG, SanSheng, Ningbo, China) followed by 10 IU human chorionic gonadotropin (hCG, ShuSheng, Ningbo, China) ~ 48 h later. For in vivo embryo collection, super-ovulated female mouse was caged individually with one fertile male overnight. 13 h post-hCG (phCG) injection, mating was confirmed by the presence of the vaginal plug. Embryos were obtained by flushing the fallopian tubes and uterine horns with M2 medium. 2-cell, 4-cell, morula and blastocyst stage embryos were collected at 42–44, 51–53, 84–86 and 92–94 h post-hCG injection, respectively. For in vitro oocytes/embryos collection, metaphase II phase (MII) oocytes were harvested from the ampulla of the oviducts at 14–16 phCG in human tubal fluid (HTF) medium. Sperm were obtained from the caudal epididymis of adult male mice and capacitated for 1.5 h in HTF medium, and the sperm were co-incubated in HTF for 2 h for in vitro fertilization. Fertilized oocytes were cultured till blastocyst stage in KSOM (MR-121-L; Millipore, Billerica, MA, USA) medium under different oxygen conditions. Zygotes were collected at 2, 4, 6, 8, and 10 h post-insemination (hpi). Embryos were collected at 24 hpi (2-cell), 48 hpi (4-cell), 72 hpi (morula) and 96 hpi (blastocyst), respectively. Embryos cultured or obtained from 4 different conditions were compared: (1) atmospheric oxygen group: in vitro fertilized oocytes were cultured in atmospheric oxygen concentration (~ 20% O_2_) from zygote to blastocyst stage; (2) in vivo group: embryos were collected by flushing oviduct and uterine horns at specific timepoint as mentioned above; (3) oxygen gradient group: in vitro fertilized oocytes were cultured at 5% oxygen concentration for 3 days and then changed to 2% oxygen concentration; and (4) hypoxia group: in vitro fertilized oocytes were cultured in 2% oxygen concentrations from zygote to blastocyst stage. The inhibitor GSK2837808A (GSKA) was dissolved in DMSO and added to KSOM medium to inhibit lactate dehydrogenase A (LDHA) activity. As a control, embryos were incubated with the matching concentration of DMSO. The developmental potential of 4-cell, morula and blastocyst stage embryos was assessed morphologically under a Leica dissection microscopy at 48 hpi, 72 hpi and 96 hpi, respectively.

### Immunofluorescence staining and quantification of fluorescence intensity

The collected oocytes/embryos were fixed in phosphate-buffered saline (PBS) containing 4% (w/v) paraformaldehyde (PFA) for 20 min at room temperature, and washed three times with 1% BSA/PBS. Then, the cells were permeabilized with 0.5% Triton X-100, 1% BSA/PBS for 20 min, and washed three times with 1% BSA/PBS. The samples were blocked with 0.1% Triton X-100, 1% BSA/PBS for 30 min and incubated with primary antibodies overnight at 4 °C. Oocytes/embryos were incubated with secondary antibodies for 1 h in dark and washed with 1% BSA/PBS for 5 min, three times. Then the samples were stained with 4,6-diamidimo-2-phenylindole (DAPI) for 10 min. Finally, the samples were mounted on slides and immunofluorescence images were taken under a Carl Zeiss LSM710 laser scanning confocal microscopy. Semi-quantitative analysis of fluorescence signals was conducted using Image J, ROI Manager Tool. The cytoplasmic pixel value of the protein was subtracted from the nucleus pixel value of the protein as the fluorescence intensity value.

### Antibodies

Primary antibodies and the dilution: anti Pan-Kla (PTM-1401, PTM Bio) 1:500, anti-H3K23la (PTM-1413, PTM Bio) 1:500, anti-H3K23ac (ab177275, Abcam) 1:500, anti-H3K18la (PTM-1406RM, PTM Bio) 1:200, anti-H3K18ac (PTM-0158, PTM Bio) 1:200, anti-HIF1α (D1S7W, Cell Signaling Technology) 1:200, anti-HIF2α (NB-100–122,) 1:200. Secondary antibodies and the dilution: anti-rabbit IgG Fab2 Alexa Fluor (R) 488 Molecular Probes (Cell Signaling Technology) 1:600; DyLight 594-conjugated AffiniPure Goat Anti-Rabbit IgG (Jackson IR) 1:100.

### Real-time quantitative PCR

RT-qPCR was done as previously reported [60]. Briefly, twenty blastocysts were dissolved in RNAiso Plus (Takara, Japan) and stored at − 80 °C till use. Total RNA was isolated from blastocysts following the manufacturer's instructions and reverse transcribed to cDNA using PrimeScript™ RT Reagent Kit with gDNA Eraser (Takara, Japan). cDNA was used as template for quantitative PCR with TB Green ®Premix EX Taq TM (Takara, Japan). 12.5 μL reaction solution composed of 1 μL cDNA, 6.25 μL TB Green Premix TaqII (2×), 0.4 μL of each primer and 4.45 μL ddH_2_O was incubated in LightCycler480 real-time PCR system (Roche, Switzerland) and amplified using two-step conditions: 95 °C for 30s; 40 cycles of 95 °C for 5s and 60 °C for 20s. All amplifications were done in technical duplicate and biological triplicate and data were analyzed using LightCycler 96 SW 1.1 software. 18s rRNA was used as the internal control and relative expression levels of the target genes were calculated by 2^−ΔΔCT^ method. The primer sequences for the LDHA, standard 18s rRNA are listed as below: LDHA sense: 5′-TGT CTC CAG CAA AGA CTA CTG T-3ʹ; LDHA anti-sense: 5′-GAC TGT ACT TGA CAA TGT TGG GA-3ʹ; 18s rRNA sense: 5′-CTG CCC TAT CAA CTT TCG ATG GTA G-3ʹ; 18s rRNA anti-sense: 5′-CCG TTT CTC AGG CTC CCT CTC-3ʹ.

### UPLC–ESI–MS analysis

After washing with PBS, the cells were repeatedly frozen and thawed in liquid nitrogen and 37 °C water bath to extract intracellular lactate. The samples were analyzed by UPLC-QQQMS, and separated by an Agilent ZORBAX SB-C18 RRHT column (50 × 2.1 mm i.d.; particle size 1.8 μm) at 30 °C. The mobile phase was composed of solvent A, 0.1% formic acid in H_2_O, and solvent B, 100% MeOH. The gradient elution was programmed as follows: 0 min, 10% B; 3 min, 100% B; 4 min, 100% B and Post time, 2.0 min. The flow rate was kept at 0.3 ml/min and the injection volume was 3 μL. For quantification of the lactate, multiple reactions monitoring (MRM) mode was used with negative polarity. The MRM conditions were optimized using L-lactate standards. The MW (90), Precursor ion (89), Product ion (43.2), Fragmentor (50), and Collision energy (10) were kept constant throughout the analysis. For ESI, the ion source, parameters were set as below: capillary, 4 kV; gas temperature, 350 °C; gas flow, 11 L/min; nebulizer, 45 psi.

### Statistical analysis

All experiments were repeated at least three times and results were expressed as mean ± SEM (standard error of the mean). More than 30 blastomeres were analyzed in each replicate of fluorescence intensity quantification and more than 20 embryos were analyzed in each replicate for the assessments of embryonic development and relative gene expression. Unpaired Student’s *t* test was used for two-group comparisons. For comparison between multiple groups, two-way analysis of variance (ANOVA) was performed followed by Tukey's multiple comparisons test [61]. The data were analyzed by GraphPad Prism version 7 (GraphPad Software, La Jolla, CA, USA). *p* values less than 0.05 was used to determine significant difference. *p < 0.05, **p < 0.01, ***p < 0.001 and ****p < 0.0001.

## Supplementary Information

**Additional file 1.** Figure S1.

**Additional file 2.** Figure S2.

**Additional file 3.** Figure S3.

**Additional file 4.** Figure S4.

## Data Availability

The data used and/or analyzed during the current study are available from the corresponding author on reasonable request.

## Abbreviations

PTMs: Posttranslational modifications
Kla: Lysine lactylation
HIFs: Hypoxia Inducible Factors
LDHA: Lactate dehydrogenase A
H3K23la: Histone H3 lysine23 lactylation
GV: Germinal vesicle
MII, Metaphase II: Hpi, hour post-insemination
GSKA: GSK2837808A
IVF: In vitro Fertilization
PBS: Phosphate-buffered saline
PFA: Paraformaldehyde
DAPI: 4′,6-Diamidino-2-phenylindole
PMSG: Pregnant mare serum gonadotropin
HCG: Human chorionic gonadotropin
