# Supplementary material for: Hypoxic in vitro culture reduces histone lactylation and impairs pre-implantation embryonic development in mice
Source: Epigenetics Chromatin. 2021 Dec 21;14:57. doi: 10.1186/s13072-021-00431-6 (PMC8691063; doi:10.1186/s13072-021-00431-6)
Supplement: Supplementary file 1 — Additional file 1. Figure S1. [file 13072_2021_431_MOESM1_ESM.pdf]

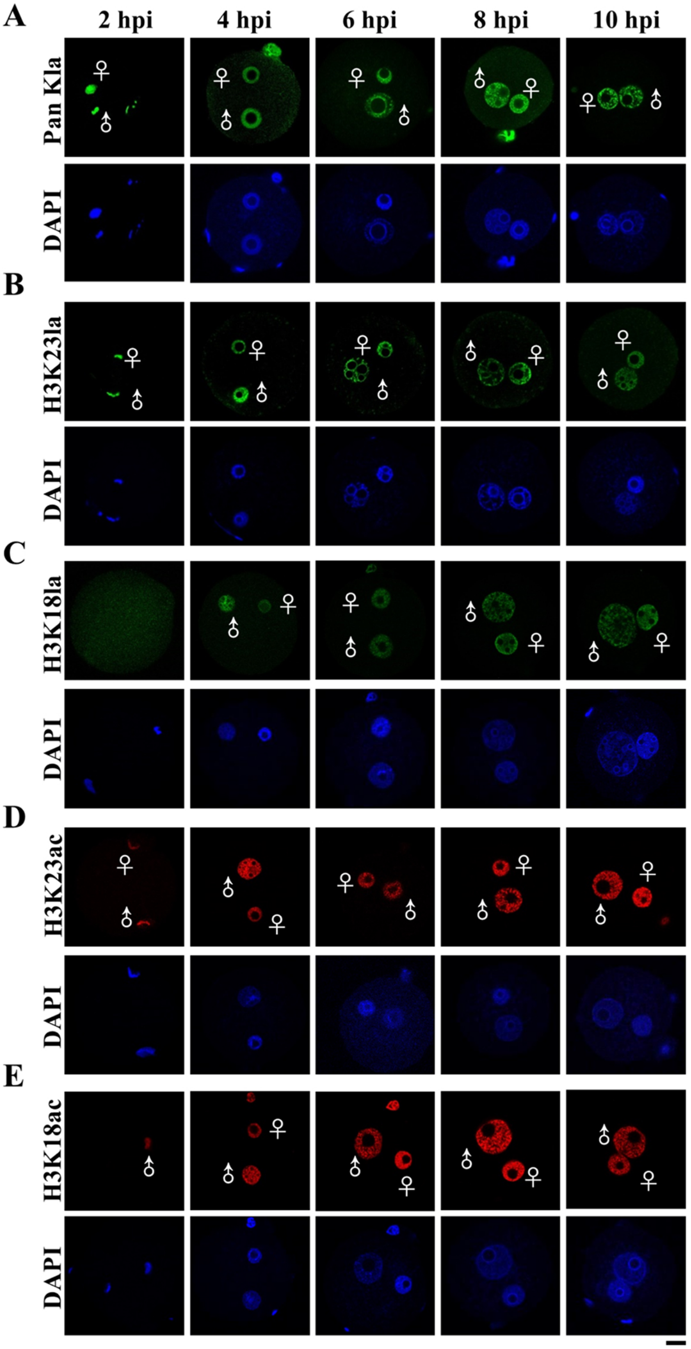

Figure S1 Nuclear accumulation of histone lactylation and histone acetylation in mouse zygotes

a-e Zygotes at 2, 4, 6, 8 and 10 hpi were immunostained with antibodies against pan histone lactylation (**a**), H3K23la (**b**), H3K18la (**c**), H3K23ac (**d**) and H3K18ac (**e**). Pan histone lactylation, H3K23la and H3K18la were shown in green. H3K23ac and H3K18ac were shown in red. The DNA was stained with DAPI (Blue). More than 15 embryos were examined in each stage each conditon. ♀ Female pronucleus; ♂ Male pronucleus. Scale bars: 20 μm.
