# Supplementary material for: Hypoxic in vitro culture reduces histone lactylation and impairs pre-implantation embryonic development in mice
Source: Epigenetics Chromatin. 2021 Dec 21;14:57. doi: 10.1186/s13072-021-00431-6 (PMC8691063; doi:10.1186/s13072-021-00431-6)
Supplement: Supplementary file 2 — Additional file 2. Figure S2. [file 13072_2021_431_MOESM2_ESM.pdf]

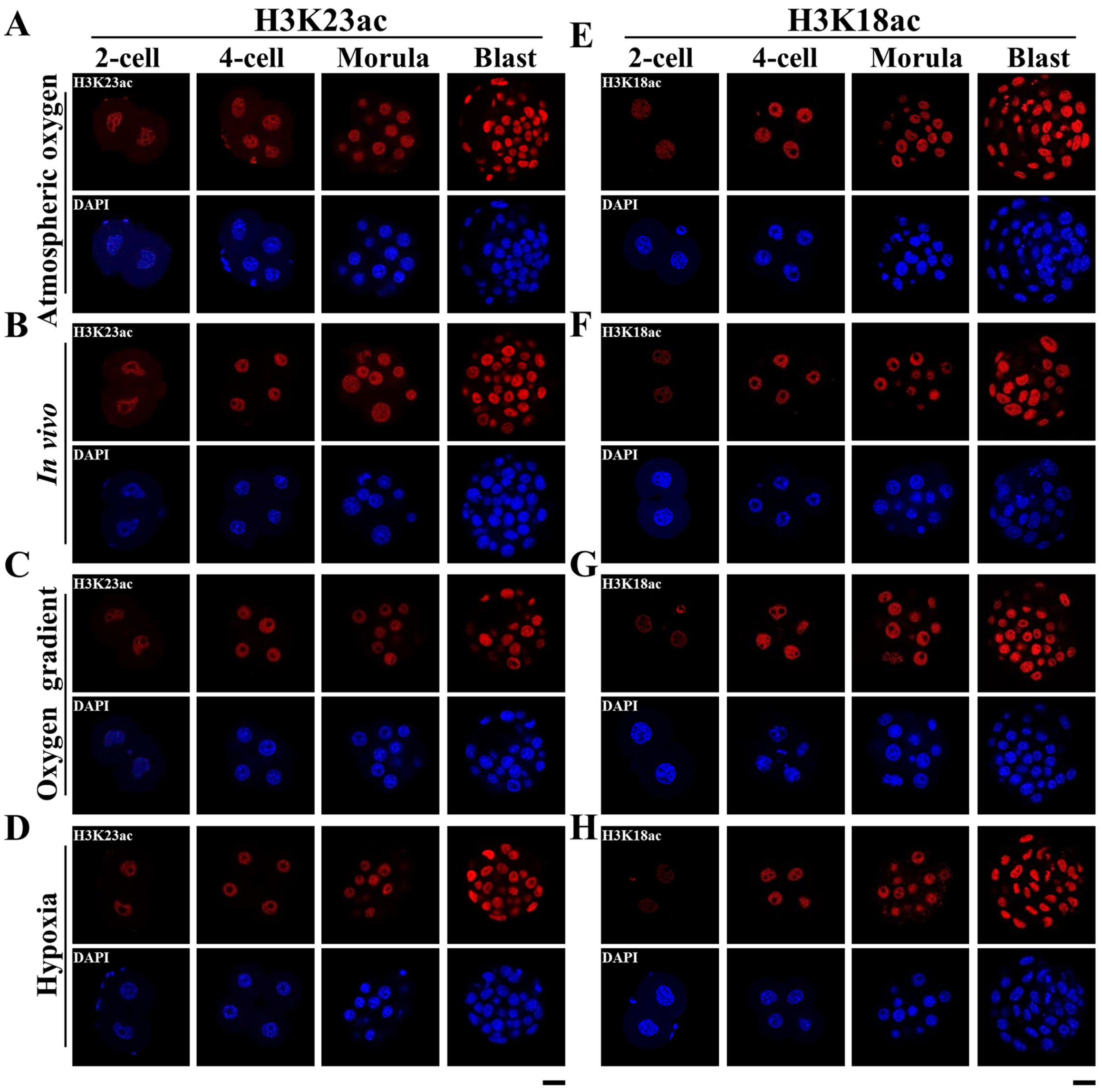

Figure S2 Nuclear accumulation of H3K23ac and H3K18ac in pre-implantation embryo under different oxygen concentrations.

a-d Immunofluorescence staining for H3K23ac (Red) in mouse embryos at the 2-cell (2-cell), 4-cell (4-cell), morula (Morula) and blastocyst (Blast) stages embryos collected from the atmospheric oxygen (**a**), *in vivo* (**b**), oxygen gradient (**c**) and hypoxia (**d**) groups. DNA was stained with DAPI (Blue). **e-h** Immunofluorescence staining for H3K18ac (Red) in mouse embryos at the 2-cell (2-cell), 4-cell (4-cell), morula (Morula) and blastocyst (Blast) stages embryos collected from the atmospheric oxygen (**e**), in vivo (**f**), oxygen gradient (**g**), and hypoxia (**h**) groups. DNA was stained with DAPI (Blue). More than 9 embryos were examined in each stage each conditon. Scale bars: 20 μm.
