# Supplementary material for: Hypoxic in vitro culture reduces histone lactylation and impairs pre-implantation embryonic development in mice
Source: Epigenetics Chromatin. 2021 Dec 21;14:57. doi: 10.1186/s13072-021-00431-6 (PMC8691063; doi:10.1186/s13072-021-00431-6)
Supplement: Supplementary file 3 — Additional file 3. Figure S3. [file 13072_2021_431_MOESM3_ESM.pdf]

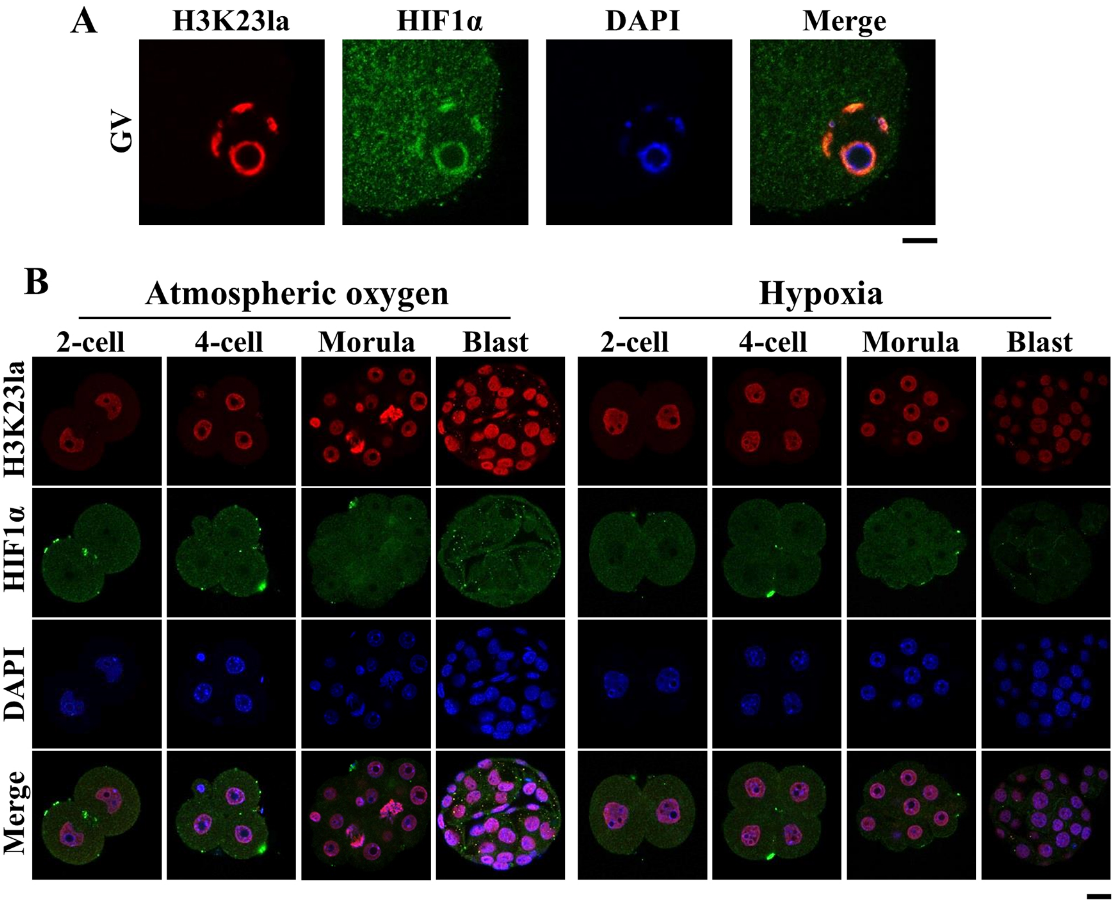

Figure S3 Nuclear localizations of HIF1α in mouse oocytes and pre-implantation embryos. **a** Oocytes at the germinal vesicle (GV) were immuno-stained simultaneously with antibodies against H3K23la (Red) and HIF1α (Green). **b** HIF1α was almost undetectable in early pre-implantation embryos. Immunofluorescence staining for H3K23la (Red) and HIF1α (Green) in pre-implantation embryos at the 2-cell (2-cell), 4-cell (4-cell), morula (Morula) and blastocyst (Blast) stages obtained from the atmospheric oxygen group and the hypoxia group. DNA was stained with DAPI (Blue).). More than 10 embryos were examined in each stage each condition. Scale bars: 20 μm.
