# Supplementary material for: Hypoxic in vitro culture reduces histone lactylation and impairs pre-implantation embryonic development in mice
Source: Epigenetics Chromatin. 2021 Dec 21;14:57. doi: 10.1186/s13072-021-00431-6 (PMC8691063; doi:10.1186/s13072-021-00431-6)
Supplement: Supplementary file 4 — Additional file 4. Figure S4. [file 13072_2021_431_MOESM4_ESM.pdf]

A

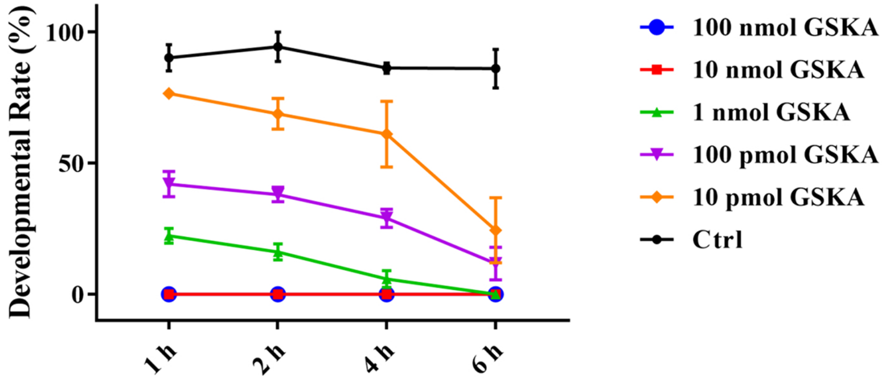

Figure S4 Assessment of the developmental inhibitory effect of GSKA

a Morula stage embryos (72 hpi) were cultured with 100 nmol, 10 nmol, 1nmol, 100 pmol, or 10 pmol of GSKA for 1, 2, 4 and 6 hours, respectively. The embryos were then transferred into drug-free medium to culture till blastocyst stage. Blastocyst developmental rates were determined relative to the staring morula stage embryo numbers (more than 120 morula stage embryos obtained from 3 independent experiments were tested in each group). Error bars indicates SEM.
